# Editorial: Tools and strategies that promote the return to work and stay at work of people with disabilities—contributions from occupational therapy and other disciplines centered on the biopsychosocial approach

**DOI:** 10.3389/fresc.2023.1293467

**Published:** 2023-10-12

**Authors:** Edda Capodaglio, Enrico Oddone

**Affiliations:** ^1^ Clinical Scientific Institute Maugeri IRCSS, Occupational Therapy and Ergonomics Unit, Pavia, Italy; ^2^ Department of Public Health, Experimental and Forensic Medicine, University of Pavia, Pavia, Italy

**Keywords:** people with disabilities, return to work, functional assessment, ICF (International Classification of Functioning Disability and Health), workplace adaptations

**Editorial on the Research Topic** Tools and strategies that promote the return to work and stay at work of people with disabilities. contributions from occupational therapy and other disciplines centered on the biopsychosocial approach

Occupational therapists are very close to patients undergoing the final rehabilitation phase. They contribute to the understanding of the consequences of disability, to the identification of resources and recognition of critical issues, and support the patient to adapt to the working, leisure, and daily environments, structuring activities and finding equipment, in order to improve his/her participation and quality of life. Inclusive and accessible working environments and activities are an important basis for the integration of people with disabilities by supporting independence and participation in daily life.

Defining and quantifying parameters relating to disability is crucial (1) and imposes the need to develop dedicated, internationally tested measurement tools. These tools would be able to detect not only the actual employment conditions but also the employability of people with disabilities (i.e., their ability to be employed and to seek, find, and keep a job). Overall, although the concept of participation has gained widespread acceptance in the rehabilitation context, its operationalization and measurement remain difficult. Complicating the measurement is the subjective nature of participation and the complex relationships with environmental factors (2).

This Research Topic includes three original research studies and one systematic review, all dealing with important topics such as the functional assessment of the person with disability and the interventions that can help increase the rate of return to work (RTW) after disability.

The need to broaden the vision related to the condition of disability emerges clearly from these works, which consider the person's abilities, psychological, and environmental factors. Immediate reference to the ICF (International Classification of Functioning Disability and Health) (3), with its comprehensive set of categories of information rendered through a unified and coherent language of human functioning, is suitable for comparing health information, in order to

- evaluate the right to sickness compensation (“disability eligibility determination”), in association with the information deduced from occupational sickness certificates which are available to occupational doctors (Fresk et al.), or in association with the evaluation of the Activity-Participation categories (FUNDES questionnaire 7.0, derived from WHODAS 2.0, to be used in clinical-outpatient settings) (Liao et al.);
- design rehabilitation paths or professionalizing interventions (“vocational rehabilitation”) aimed at RTW (Fresk et al.) and inform disability management strategies aimed at increasing the employability rate (Thygesen et al.);
- evaluate the functioning and limitations of the individuals in greater detail, expanding the concept of workability, as a screening/evaluation tool for work participation (Thygesen et al.);
- provide useful data to support the planning of services for the disabled population, i.e. public transport (Liao et al.);
- provide data for the classification of disability on the basis of functioning (not only on the basis of body structures and functions) (Liao et al.);
- detect personal and environmental determinants that can help plan interventions for the prevention of work disability and for minimizing the impact of disability (Duong et al.).

The Topic Authors note some shortcomings of the ICF (i.e., the lack of consideration about the effects due to the intake of drugs/analgesics/opioids, or about the effect related on social support), as well as suggest the integration of missing on psychological and socio-economic factors (i.e., focusing attention, sleep, memory, interpersonal relations, remunerative employment).

Other authors report even greater uncertainty about ICF, considering its irrelevancy in understanding the mutual and complex relationships between environmental factors and individual, community, and social levels (4, 5). These authors claim the role of the environment as a “scene setter” for disability, and not just as a facilitator or barrier, particularly in relation to the performance of roles, tasks, and social involvement.

Another use of ICF, not covered in this Research Topic but reported in the literature (6), concerns the design and evaluation of interventions aiming at “workplace reasonable adaptations.” These interventions prevent long-term disability by acting on the exposure to occupational risk factors (i.e., working time, work-flow organization, posture, strength, frequency of action, environmental factors). It is worth taking into account this perspective, as occupational risk assessment systems traditionally apply to a population of “healthy” workers, while exposure thresholds for workers with disabilities are unknown. Since people with disabilities show high variability in terms of ability, use of a standard method (ICF qualifiers) referring to actual “performance” could help hypothesize the best placement of the worker suffering from any form of deficit affected by disability.

ICF performance level specifications, supported by assistive devices or a technologically equipped environment, could help prepare suitable activities and workstations. The ICF framework served well in providing a foundation for understanding issues of functioning and disability, and could assist in developing an optimized re-design of the workplace that enables the worker to continue the same activities at an appropriate level of productivity and comfort, without any risk (7). The need to include environmental changes in the planning of RTW and stay at work means proactively supporting the participation of people with disabilities at the community or societal levels.

This Research Topic highlights strengths associated with the use of ICF in designing pathways for RTW such as

- the increase in the discretionary capacity of doctors and clinicians in first contact with workers with disabilities; in this regard, the figure of the Occupational Therapist should be valued too, above all in the sense of the opportunity to conduct a targeted assessment in the workplace;
- the broadening of the vision of disability, exploring the reasons that potentially distance the subject from work.

Finally, there are some issues which will need to be addressed by research, such as RTW's definition, use of validated prognostic factors, disability in low-income countries, pathology-specific ICF Core-Sets (i.e., musculoskeletal disorders), and studies related to mental health (8) as it is associated with precarious and atypical forms of work (9) or exceptional events such as the pandemic.
